# Supplementary material for: The interactome of intact mitochondria by cross-linking mass spectrometry provides evidence for coexisting respiratory supercomplexes
Source: Mol Cell Proteomics. 2017 Dec 8;17(2):216–32. doi: 10.1074/mcp.RA117.000470 (PMC5795388; doi:10.1074/mcp.RA117.000470)
Supplement: Supplemental Data [file supp_RA117.000470_133922_0_supp_23978_fzffwf.zip › spectra_annotation/mito_DR_spectra_annotation/448-1-4-1-5-1.pdf]

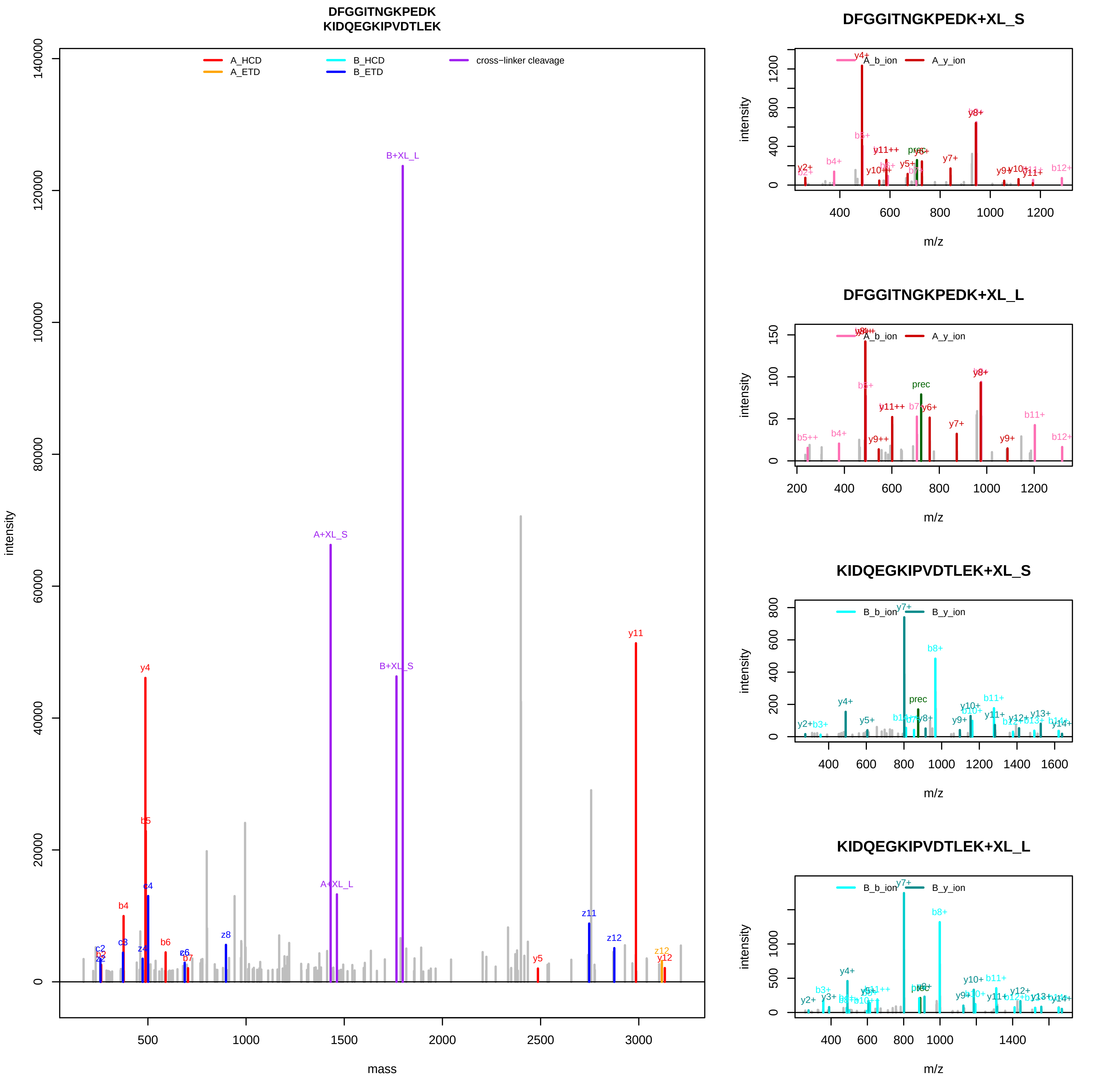

DFGGITNGKPEDK
KIDQEGKIPVDTLEK
A_HCD
A_ETD
B_HCD
B_ETD
cross−linker cleavage
B+XL_L
A+XL_S
B+XL_S
A+XL_L
y4
b5
b4
c4
c2
c3
z4
b6
z6
b7
z8
y5
z11
z12
y11
y12
intensity
mass
DFGGITNGKPEDK+XL_S
DFGGITNGKPEDK+XL_L
KIDQEGKIPVDTLEK+XL_S
KIDQEGKIPVDTLEK+XL_L
A_b_ion
A_y_ion
B_b_ion
B_y_ion
prec
m/z
